# Compatibility of Sustainable Mater-Bi/poly(ε-caprolactone)/cellulose Biocomposites as a Function of Filler Modification

**DOI:** 10.3390/ma16206814

**Published:** 2023-10-23

**Authors:** Aleksander Hejna, Mateusz Barczewski, Paulina Kosmela, Olga Mysiukiewicz, Adam Piasecki, Agnieszka Tercjak

**Affiliations:** 1 Institute of Materials Technology, Poznan University of Technology, Piotrowo 3, 61-138 Poznań, Poland; mateusz.barczewski@put.poznan.pl (M.B.); olga.mysiukiewicz@put.poznan.pl (O.M.); 2 Department of Polymer Technology, Gdańsk University of Technology, Narutowicza 11/12, 80-233 Gdańsk, Poland; paulina.kosmale@pg.edu.pl; 3 Institute of Materials Engineering, Poznan University of Technology, Piotrowo 3, 61-138 Poznań, Poland; adam.piasecki@put.poznan.pl; 4 Group ‘Materials + Technologies’ (GMT), Department of Chemical and Environmental Engineering, Faculty of Engineering Gipuzkoa, University of the Basque Country (UPV/EHU), Plaza Europa 1, 20018 Donostia-San Sebastian, Spain; agnieszka.tercjak@ehu.eus

**Keywords:** Mater-Bi, poly(ε-caprolactone), cellulose filler, biocomposites, filler modification, interfacial adhesion

## Abstract

Despite their popularity and multiplicity of applications, wood–polymer composites (WPCs) still have to overcome particular issues related to their processing and properties. The main aspect is the compatibility with plant-based materials which affects the overall performance of the material. It can be enhanced by strengthening the interfacial adhesion resulting from physical and/or chemical interactions between the matrix and filler, which requires introducing a compatibilizer or a proper modification of one or both phases. Herein, the impact of cellulose filler modifications with varying contents (1–10 wt%) of hexamethylene diisocyanate (HDI) on the compatibility of Mater-Bi/poly(ε-caprolactone) (PCL)-based biocomposites was evaluated. An analysis of surface wettability revealed that the filler modification reduced the hydrophilicity gap between phases, suggesting compatibility enhancement. It was later confirmed via microscopic observation (scanning electron microscopy (SEM) and atomic force microscopy (AFM)), which pointed to the finer dispersion of modified particles and enhanced quality of the interface. The rheological analysis confirmed increased system homogeneity by the reduction in complex viscosity. In contrast, thermogravimetric analysis (TGA) indicated the efficient modification of filler and the presence of the chemical interactions at the interface by the shift of thermal decomposition onset and the changes in the degradation course.

## 1. Introduction

Wood–polymer composites (WPCs) are a group of composite materials consisting of one or more plant-based fillers and a mixture of polymers. Over the last decades, the research works on their development aim to address the main problems related to their processing: (i) the variations in the quality of plant-based materials; (ii) the limited thermal stability of plant-based materials narrowing the processing window for WPCs; and mostly, (iii) the limited compatibility often between the hydrophobic polymer matrix and hydrophilic fillers [1,2,3,4,5].

Insufficient compatibility is a critical issue as strong interfacial interactions are crucial for achieving the good mechanical properties of composites [6]. Enhancing the compatibility may be realized by providing possibilities for the chemical bonding of modified filler with the functional groups present in the polymer backbone or by changing the character of the fillers’ surface from hydrophilic to hydrophobic, simultaneously enhancing the filler and matrix mutual affinity. It often requires proper modifications of introduced fillers. Over the last decades, multiple review works related to their chemical modification, mainly aimed at enhancing the performance of WPCs, have been published by different research groups [7,8,9,10,11,12]. The authors mentioned numerous filler modifications, of which the most popular and widely described are alkali treatment, silanization, acetylation, maleation, acrylation, or permanganate treatment. Less common modification methods include fatty acid modification, sodium chloride treatment, benzoylation, triazine, or isocyanate treatment. Collectively, their goal was to provide the possibility for covalent bonding with a polymer matrix or to reduce the hydrophilicity gap between phases.

Generally, the most popular modifiers of lignocellulosic fillers are anhydrides such as maleic anhydride, succinic anhydride, dodecenyl succinic anhydride, or phtalic anhydride [13,14]. These compounds are often present in biocomposite manufacturing because they can be grafted onto polymer chains, creating strong covalent bonds with hydroxyl groups on the lignocellulosic fillers’ surface. By determining the proper choice of anhydride (molecular weight, potential steric hindrance), the properties of modified filler, e.g., crystallinity or thermal stability, can be adjusted [15].

From the technological point of view, isocyanates offer similar possibilities for the adjustment of modifiers’ structure and are commonly used in the plastics industry. Hence, they are well known and analyzed. They can create urethane bonds with hydroxyls on the surface of lignocellulosic fillers yielding strong covalent bonding at the interface. The broad isocyanate market offering multiple grades with various structures and properties enables many potential modifications of lignocellulosic fillers. It has been proven, e.g., by Zhang et al. [15], that the modifier’s structure significantly influences the properties of modified filler. The most crucial properties are the molecular weight, functionality, and spatial structure. These modifier properties affect the structure of modified lignocellulosic fillers, e.g., its crystallinity, which has an essential impact on the mechanism and rate of lignocellulosic fillers’ and composites’ biodegradation. Therefore, isocyanates seem an auspicious solution for the enhancement of interfacial interactions.

A contentious issue always raised in the case of isocyanates’ application is their toxicity. They are considered toxic due to highly reactive isocyanate groups in their structure [16]. Nevertheless, isocyanate is bonded and reacted with other functional groups, e.g., hydroxyl or amine groups, and no toxic effects are observed. Therefore, it is possible to use polyurethane materials to manufacture biomedical-grade articles, such as vascular catheters, blood bags, or implants [17]. As mentioned before, during modification of lignocellulosic fillers, isocyanate groups can create urethane bonds with the hydroxyls present on the surface of fillers, so the toxic effect should be significantly reduced due to the excess of hydroxyl groups of fillers.

As a group, isocyanates have been repeatedly proven as excellent coupling agents for polyolefin-based biocomposites, revealing properties superior to those compatibilized with anhydrides [18,19]. Various research groups analyzed lignocellulosic fillers’ modification with isocyanates and further processing of biocomposites at temperatures from 150 to 175 °C, which enhanced composites’ mechanical performance. It is also important to mention that the authors of these works used periodic methods. Therefore, the time of modification and the influence of temperature on lignocellulosic fillers was longer than continuous methods. Despite that, no material degradation was observed [20,21].

Liew et al. [22] analyzed the hexamethylene diisocyanate (HDI) in compatibilization of low-density polyethylene composites filled with jute and bamboo fibers. The authors applied a periodic method of fillers’ modification, which included the incorporation of solvents and an additional 24 h process of drying, which negatively impacted the filler’s modification cost. Nevertheless, modification of lignocellulosic fibers with HDI noticeably enhanced the thermal stability of the composite containing 10 wt% of fillers, shifting the onset of thermal decomposition by more than 100 °C, which could be a great advantage from the technological point of view. It would allow using such modified fillers to manufacture composites based on polymers with higher processing temperatures. Moreover, isocyanate treatment of fillers significantly enhanced interfacial adhesion between the matrix and filler, which was proven via SEM analysis, and increased the tensile strength and Young’s modulus by 295 and 296%, respectively.

Gómez-Fernández et al. [16] investigated lignin modification with isophorone diisocyanate (IPDI). The authors presented the functionalization of kraft lignin in a periodic method, using dibutyltin dilaurate as a catalyst for the reactions between the isocyanate groups of IPDI and the hydroxyl groups present on the surface of lignin. The method involved conducting a chemical reaction at 60 °C for 24 h, further centrifugation, washing in toluene, drying for another 24 h, and grinding the obtained particles. Functionalization of lignin was confirmed via a detailed analysis of the structure and composition of the filler with FTIR, liquid, solid-state NMR spectroscopy, and elemental analysis. Moreover, modification decreased the average particle size, which can be considered very beneficial from the application point of view. Nevertheless, despite the successful modification, the presented procedure has noticeable disadvantages: extended time, complex character (multiple processes involved), and the use of organic solvents.

A less popular direction is the application of isocyanates for the compatibilization of biocomposites based on sustainable polymer matrices. The application of isocyanates has been analyzed rather in terms of the compatibilization of blends, modified pea starch and dextrin [23], poly(ε-caprolactone) (PCL) and starch [24,25], or poly(lactic acid) with poly(butylene succinate) [26]. Filler modification with isocyanates aimed to enhance polyolefin-based composites [22,27,28]. Our previous work [29] showed that the PCL-based biocomposites’ static and dynamic mechanical performance could be significantly boosted by appropriately modifying cellulose filler with diisocyanates by enhancing interfacial adhesion. Interestingly, diisocyanate modifications of cellulose filler also affected the biodegradation rate of the prepared composites [30]. A similar phenomenon has been noted for Mater-Bi/brewers’ spent grain composites containing IPDI-modified filler [31,32].

Having in mind the results of the abovementioned works pointing to the beneficial impact of diisocyanates on the compatibility of PCL/thermoplastic starch (TPS) blends as well as the PCL- and Mater-Bi-based biocomposites, the presented work aimed to investigate the effect of the modification of cellulose filler with HDI on the structure and performance of composites based on the Mater-Bi/PCL matrix. The application of such blends as the matrix and incorporation of PCL was related to the relatively poor performance of Mater-Bi, which limits its application range [33,34,35,36,37].

## 2. Materials and Methods

### 2.1. Materials

Two polymer materials in the presented study have been applied as matrices for the composites as follows: poly(ε-caprolactone) (PCL) and Mater-Bi type NF803 composed of poly(butylene adipate-co-terephthalate) (PBAT), TPS, and PCL. Details regarding their characteristics are summarized in Table 1.

**Figure 1:**
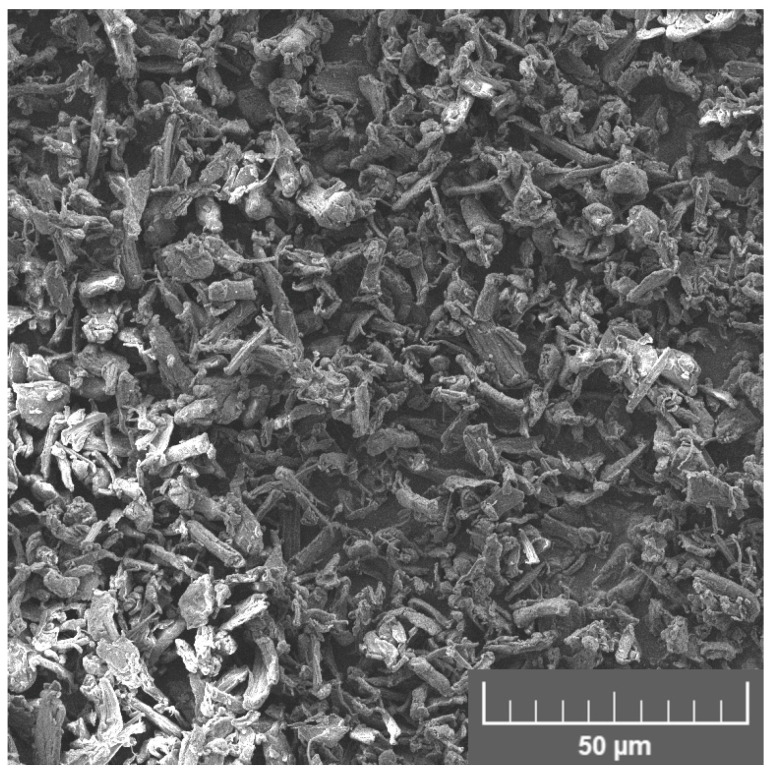
SEM image of as-received UFC100 cellulose filler.

Prepared composites were filled with cellulose micrometric particles—Arbocel^®^ UFC100 from JRS J. Rettenmaier & Söhne GmbH (Rosenberg, Germany). The applied filler had an average particle length of 8 µm, an aspect ratio of 4, a bulk density of 160 g/L, and a moisture content of 4.84 wt%. Its morphology is presented in Figure 1, obtained with scanning electron microscopy (SEM). It can be seen that the applied filler’s particles show a relatively rough surface, which should translate into the extended specific surface area.

The 98% hexamethylene diisocyanate (HDI) acquired from Sigma Aldrich (Poznan, Poland) was applied as a modifier for cellulose filler.

### 2.2. Sample Preparation

**Figure 2:**
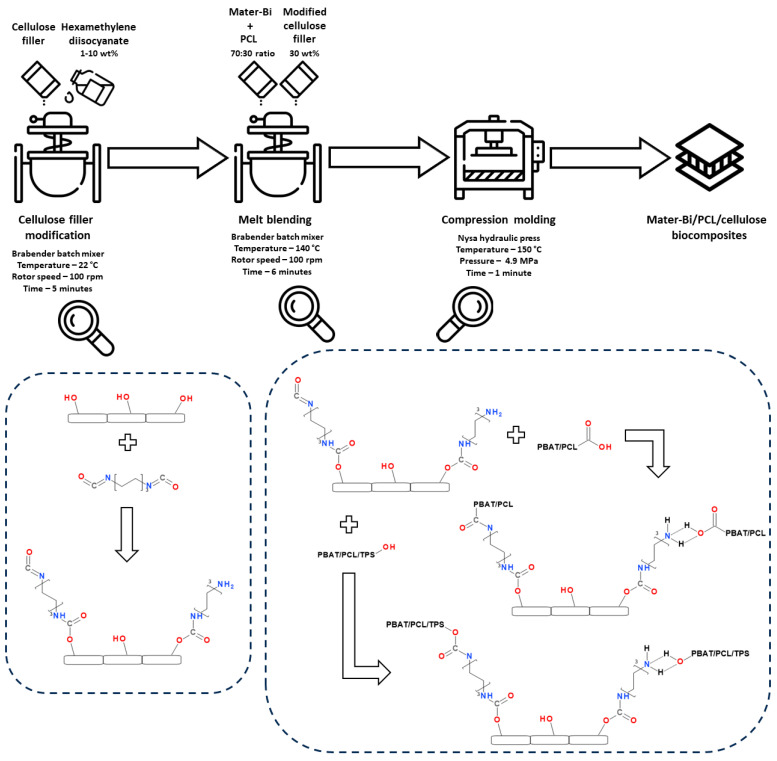
Scheme of Mater-Bi/PCL/cellulose composites preparation and potential interfacial interactions during particular processing steps.

Preparation of Mater-Bi/PCL/cellulose biocomposites included the modification of the as-received filler with 1.0, 2.5, or 10.0 wt% of HDI, melt blending with the polymer matrix, and compression molding, as schematically presented in Figure 2. The first process was described in detail in our previous works [29,38,39]. Briefly, it was conducted in a batch mixer at room temperature for 5 min using a rotor speed of 100 rpm. Further, modified fillers (30 wt%) were melt-blended with a polymer matrix comprised of Mater-Bi and PCL in a 70:30 ratio in the batch mixer. The process conducted at 140 °C and at a rotor speed of 100 rpm lasted 6 min (including the 1 min plasticization phase). Then, the materials were compression molded at 150 °C and 4.9 MPa for 1 min and kept under pressure at room temperature for 5 min to enable solidification. The unfilled Mater-Bi/PCL matrix and composite filled with the as-received filler were processed as reference samples. Figure 2 also provides the details on the potential chemical interactions between the matrix and filler during particular processing steps.

### 2.3. Characterization

Fourier transform infrared spectroscopy (FTIR) was applied to investigate the changes in the chemical structure of prepared Mater-Bi/PCL/cellulose composites caused by applying the HDI modifier. Jasco FT/IR-4600 (Tokyo, Japan) spectrometer was applied. Measurements were performed in attenuated total reflection (ATR) mode with a 4 cm^−1^ resolution from 4000 to 400 cm^−1^.

Surface wettability was studied via static water contact angle measurements using an Ossila L2004 contact angle goniometer (Ossila Ltd., Sheffield, UK) equipped with a camera and Ossila Contact Angle software v3.1.2.2. Ten water contact angle (WCA) measurements were taken in random positions, putting drops of ~1 µL distilled water onto the surface of the samples with the aid of a syringe. The average values of at least seven measurements were calculated and reported.

The surface roughness of the analyzed samples was evaluated using an ART300 surface roughness tester from Sunpoc Co., Ltd. (Guiyang, China). The sampling length was 0.8 mm. The average R_a_ value was calculated from five measurements on five different spots on the sample’s surface (25 measurements in total).

The scanning electron microscope (SEM) Tescan MIRA3 (Brno, Czech Republic) was used to analyze the brittle fracture surfaces of the prepared samples. The accelerating voltage of 12 kV was applied with a working distance of 19 mm. A thin carbon coating of approximately 20 nm thickness was deposited on the samples using the Jeol (Tokyo, Japan) JEE 4B vacuum evaporator.

Atomic force microscopy (AFM) was used to study the morphology of the prepared materials. The equipment used was a scanning probe microscope (SPM) (NanoScope IIIa Multimode from Digital Instruments, Veeco Instruments Inc., Santa Barbara, CA, USA) in tapping mode (TM-AFM). A one beam cantilever (125 mm) with a silicon probe (curvature nominal radius of 5–10 nm) was used. Samples were cut using an ultramicrotome Leica Ultracut R (Weltzar, Germany) with a diamond blade and the cross-section of each prepared material was analyzed.

Rheological investigations were carried out using an Anton Paar (Graz, Austria) MCR 301 rotational rheometer, with 25 mm diameter parallel plates and a 0.5 mm gap under the oscillatory mode. The experiments were conducted at 170 °C. The strain sweep experiments were conducted before performing the dynamic oscillatory measurements in the frequency sweep mode. The strain sweep experiments of all the samples were performed at 170 °C with a constant angular frequency of 10 rad/s in the varying strain window 0.001–100%. The preliminary investigations allow us to determine the value of the 0.05% strain as applicable for the frequency sweep experiments and locate all the samples in the linear viscoelastic region (LVE). The angular frequency used during the studies was in the range of 0.05–500 rad/s.

The thermal properties of the samples were measured via differential scanning calorimetry (DSC) carried out on a DSC 214 apparatus from Netzsch (Selb, Germany). Measurements were performed in the temperature range of −80 to 170 °C under a nitrogen atmosphere (30 mL/min gas flow) at a heating/cooling rate of 15 °C/min. The heating was performed twice to erase the thermal history of the samples. The crystallinity degree X_c_ and supercooling parameter (ΔT) were calculated using the following Equations (1) and (2):

X_c_ = ΔH_m_/((1 − ξ) × ΔH_m100%_) × 100% (1)

ΔT = T_m_ − T_c_ (2)

where ΔH_m_—sample’s melting enthalpy, J/g; ξ—weight fraction of filler; ΔH_m100%_—melting enthalpy for 100% crystalline polymer, for PCL equal to 139.5 J/g [40]; T_m_—melting temperature, °C; and T_c_—crystallization temperature, °C.

The thermal stability of materials was determined via thermogravimetric analysis (TGA) with the temperature set between 35 °C and 800 °C at a heating rate of 15 °C/min under a nitrogen flow using a TG 209 F1 Netzsch (Selb, Germany) apparatus. Samples of 10.0 ± 0.1 mg and ceramic pans were applied.

## 3. Results and Discussion

### 3.1. FTIR

**Figure 3:**
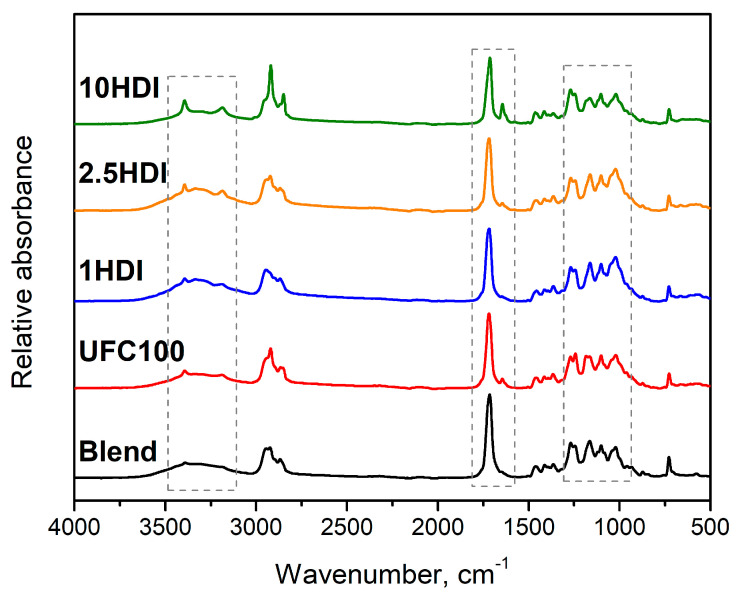
The FTIR spectra of unfilled Mater-Bi/PCL blend and prepared composites.

Figure 3 presents the FTIR spectra for the unfilled Mater-Bi/PCL blend and composites made thereof containing as-received and HDI-modified cellulose filler. Our previous works dealing with the diisocyanate modifications of UFC100 cellulose fillers revealed significant changes in their chemical structure [38,39]. The most noticeable variations in the FTIR spectra have been noted for the wavenumber ranges marked in Figure 3 with grey rectangles, which were associated with the abovementioned chemical interactions between the highly reactive free isocyanate groups of applied modifiers and the functional groups present on the surface of cellulose particles (Figure 2). These changes also affected the chemical structure of prepared biocomposites. The region most affected was from 3100 to 3500 cm^−1^, where changes in the intensity of broad O-H stretching signal were noted along with the appearance of new absorption bands around 3190 and 3400 cm^−1^. These changes in the typically broad signal can be attributed to the interactions between the HDI isocyanate groups and the hydroxyl and carboxyl groups present in the structure of polymer materials applied as a matrix, which may yield novel covalent or hydrogen bonds.

Between the wavelength range of 1610 and 1775 cm^−1^, the absorption band attributed to the stretching vibrations of carbonyl groups in the polymer structure is observed, as has been described in various works [41,42]. The introduction of cellulose filler caused a slight reduction in the signal’s intensity, which could be associated with a reduced share of the polymer matrix in the composite. However, the reduction deepened after HDI modification, which was mainly pronounced for the 10HDI sample. Such a phenomenon may point to the partial attraction of oxygen included in the carbonyl group by the NCO or NH_2_ groups of modified filler particles and its involvement in hydrogen bonding. It was also suggested by the slight peak shift toward lower wavenumbers, which is typically noted for hydrogen bonding [43]. The introduction of modified filler also caused another change in the abovementioned range—the appearance of a peak around 1644 cm^−1^, which can be attributed to the conjugation between the C-N and C=O bonds present in the urethane groups [44]. It has been previously noted in works dealing with isocyanate modification not only of cellulose [45,46], but also starch and dextrin [23] as well as castor oil [47], so it is a typical effect of chemical interactions between hydroxyl and isocyanate groups.

The abovementioned changes were also mirrored in the slight variations in the intensity of multiple weaker signals related to the vibrations of C-C, C-O, C-H, C-N, and N-H bonds typically observed in the 950–1310 cm^−1^ range.

### 3.2. Surface Wettability

**Figure 4:**
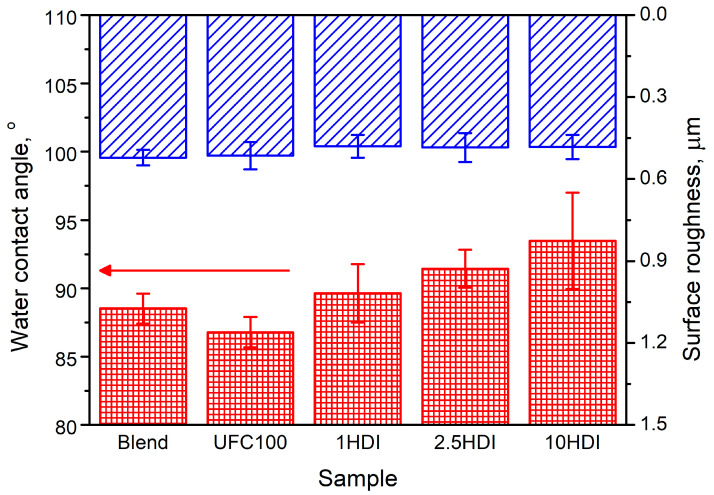
The impact of incorporating as-received and HDI-modified cellulose filler on the (red) WCA and (blue) surface roughness of composites.

As mentioned above, one of the main aspects limiting the performance of polymer composites filled with plant-based materials is attributed to the hydrophilicity difference between phases. Therefore, overcoming this issue is among the goals behind modifying cellulose fillers in the presented study. According to Ly et al. [48], the WCA of cellulose is around 47°, which is far less than ~100° reported for PCL [49], ~86° for PBAT [50], and ~95° for Mater-Bi [51,52]. As presented in Figure 4, the incorporation of unmodified cellulose filler resulted in a slight drop in the composite’s WCA from 88.5° to 86.8°, indicating a shift towards the more hydrophilic character. Similar effects have been noted by Laaziz et al. [53] after the introduction of 15 wt% argan nut shell particles into poly(lactic acid) (drop from 79.4° for unfilled PLA to 73.0°) by Tsou et al. [54], who incorporated 25 wt% of distiller’s grains into a poly(ethylene terephthalate) (drop from 91.3° to 87.0°). Such an effect can be limited by appropriately modifying the applied fillers aimed at masking their hydrophilicity. As reported in our previous work [38], modification of cellulose particles with diisocyanates leads to the changes in surface polarity expressed by the higher stability of their suspensions in less polar solvents, which enhances their compatibility with less hydrophilic or hydrophobic polymer matrices. Carvalho et al. [55] modified thermoplastic starch films with a 40% phenyl isocyanate methylene chloride solution, shifting WCA from 63° to 98°, which shows the potential of isocyanates in compatibilization with polymer composites containing plant-based fillers. In the presented case, applying 1–10 wt% of HDI led to a noticeable increase in composites’ WCA from 86.8° to 89.6–93.5°. The efficiency of isocyanates in reducing the hydrophilicity of polymer composites has also been confirmed by the results reported by Geng et al. [18], Zhang et al. [56], Liew et al. [22], and Arjmand et al. [28]. All of these works pointed to the significant reduction in the water absorption capacity of composites after the application of isocyanates as filler modifiers. Concluding, the introduction of isocyanates into polymer composites puts them on the edge of hydrophilicity and hydrophobicity, making them auspicious modifiers considering the potential biodegradation, which is strongly affected by the water absorption capacity [57,58]. Notably, all of the analyzed composites were characterized by a similar level of surface roughness, typically affecting the WCA values, pointing to the leading role of performed HDI modification in wettability changes [59,60].

### 3.3. Microstructure

**Figure 5:**
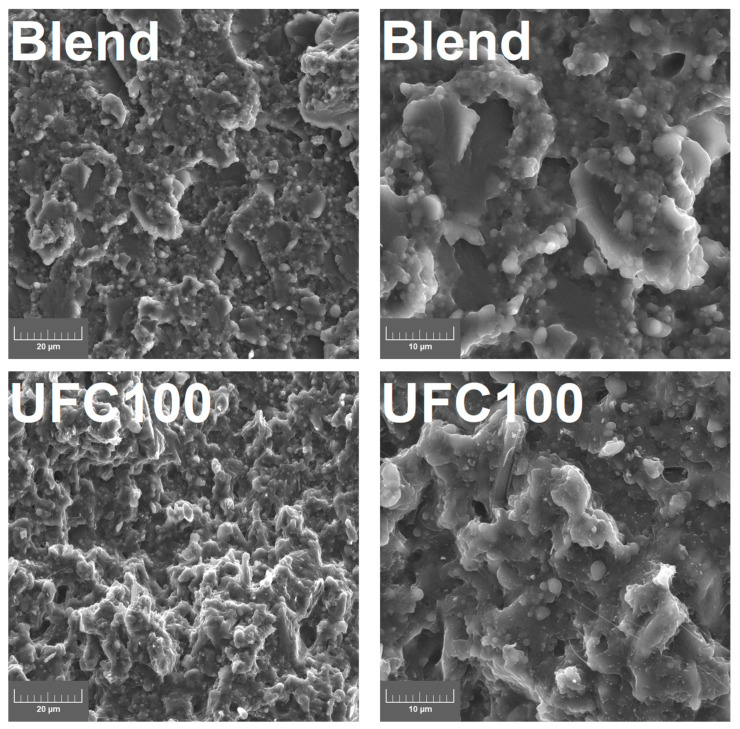

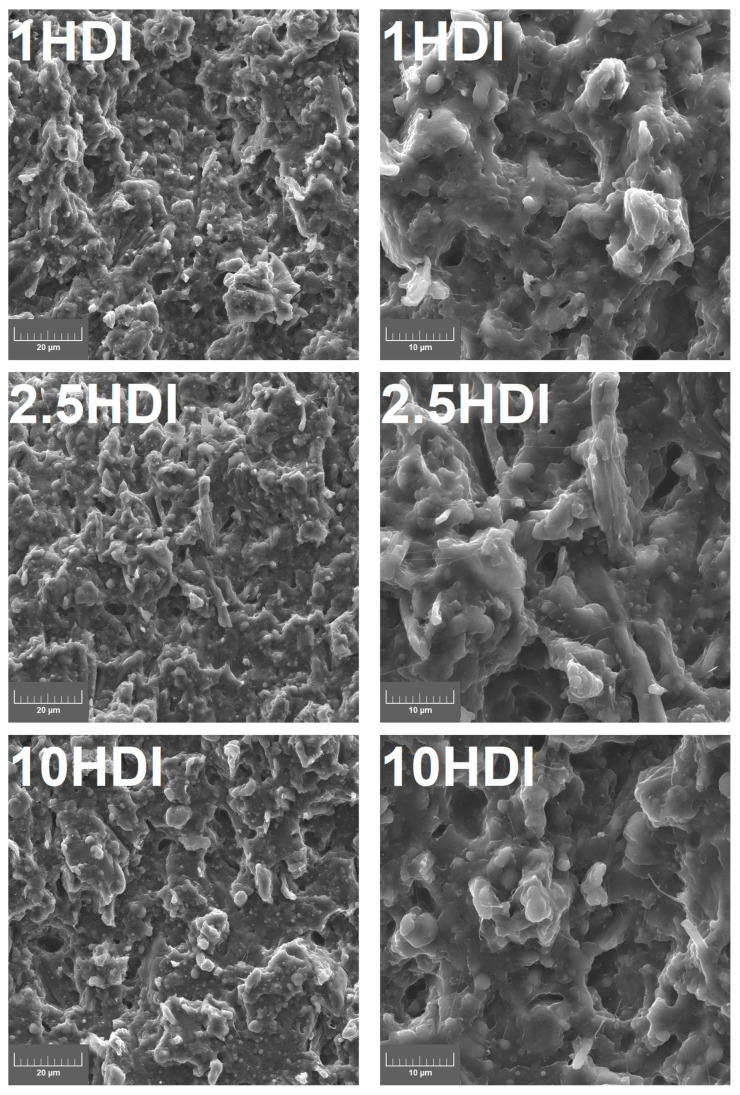
SEM images of prepared samples’ brittle fracture surfaces at different magnifications; lower (**left**) and higher (**right**).

**Figure 6:**
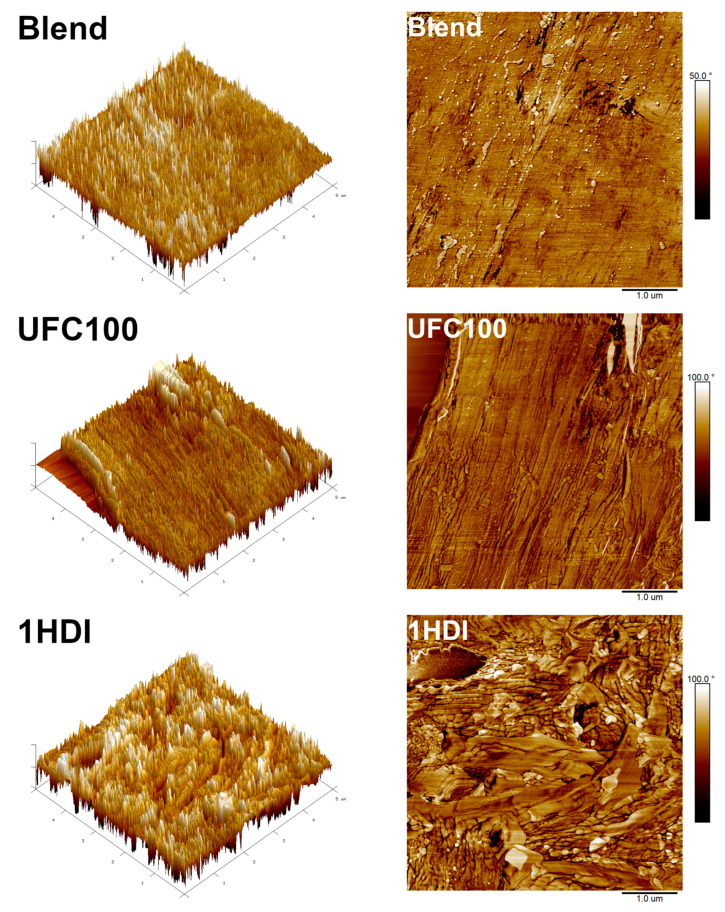

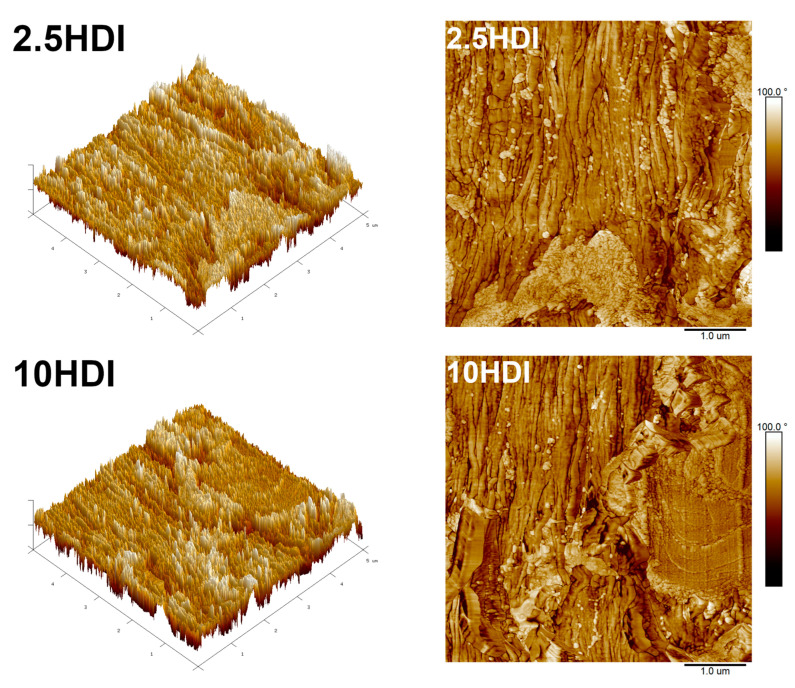
AFM phase images of the cross-sectional areas of studied samples in (**left**) 3D and (**right**) 2D mode.

Figure 5 presents the images of the prepared samples’ microstructure obtained with scanning electron microscopy. Moreover, for a deeper and more comprehensive understanding of the changes occurring at the interface after the modification of cellulose filler with HDI, AFM analysis was conducted. Figure 6, along with Table 2, provides the results of the performed analysis in qualitative and quantitative terms.

The structure of the unfilled Mater-Bi/PCL blend shows a high level of heterogeneity, pointing to the limited compatibility between particular components, PBAT, TPS, and PCL, which may be attributed to the abovementioned differences in their wettability expressed by WCA. Moreover, there are visible grain-like particles which may be attributed to the incomplete plasticization of starch and its presence in semicrystalline form, which is repeatedly noted by other researchers [51,61,62]. These particles are surrounded by the rough regions corresponding to the amorphous TPS phase. Smooth plate-shaped gaps indicate the PBAT phase, containing small PCL particles with significantly smoother surfaces than the non-plasticized starch phase [35]. AFM analysis of unfilled Mater-Bi/PCL confirmed the presence of unplasticized starch particles. Except for them, the surface of the blend was relatively smooth, especially compared to the prepared composites, which can be expressed by the lowest R_q_ and R_a_ values among all of the analyzed samples.

The introduction of unmodified UFC100 cellulose filler resulted in a significant increase in the roughness of samples’ cryofractured surfaces, which can be attributed to the shape of particles (Figure 1). AFM images indicated a noticeable increase in surface area and roughness compared to the unfilled blend, which required enhancing the magnitude of the phase shift angle. Moreover, the AFM image of the UFC100 sample points to the void resulting from the ripping out of the cellulose particle during sample preparation with ultramicrotome. Combined with the very smooth character of the interfacial area and the particles’ agglomeration and singular pull-outs indicated by the SEM images, it confirms the insufficient compatibility between the applied polymer matrix and the introduced filler resulting from the hydrophilicity gap, as suggested by the WCA values.

The modification of cellulose filler significantly enhanced the interfacial adhesion with the Mater-Bi/PCL blend, which was expressed by the noticeably limited particles’ agglomeration, as stronger interfacial interactions reduce filler aggregation [63]. Moreover, the number of pull-outs and voids was reduced, which was also confirmed by the AFM images. Sample 1HDI showed a void resulting from the filler particle ripping out, but compared to the UFC100 composite, the interface structure was noticeably different. Its roughness was noticeably higher due to the deposition of isocyanate on the filler surface and the presence of chemical structures resulting from the interactions between the matrix and the filler functional groups. The build-up of such structures was even more pronounced for the samples containing filler modified with higher HDI loadings. As a result, an interfacial area in the HDI-modified samples was characterized by negative R_sk_ values [64].

### 3.4. Rheological Behavior

**Figure 7:**
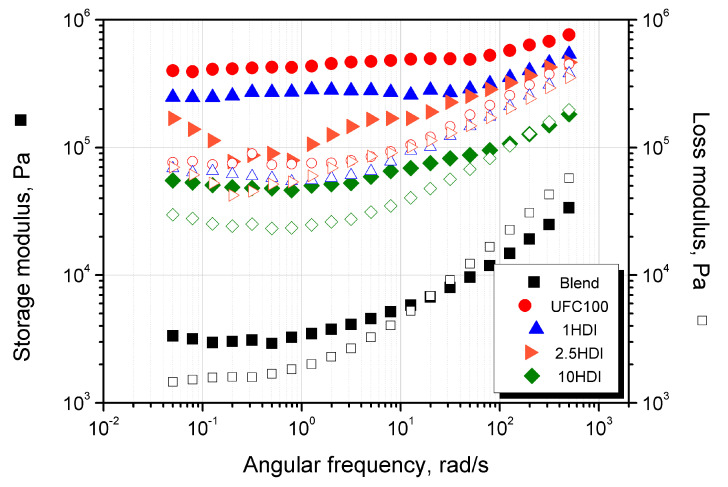
Storage (G’—■) and loss (G”—□) modulus curves as a function of angular frequency.

**Figure 8:**
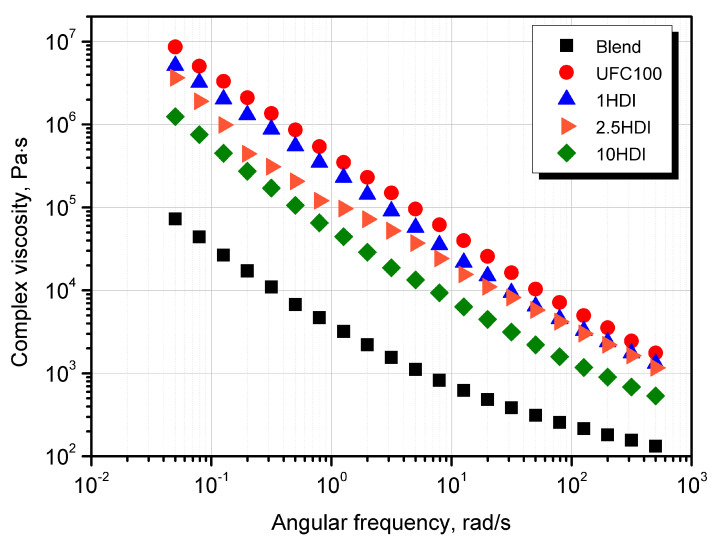
Complex viscosity of prepared materials as a function of angular frequency.

The viscoelastic behavior of polymeric composites may provide valuable information about the mutual interaction of multicomponent systems. Its detailed description may be an effective tool for qualitatively evaluating the compatibility between polymeric blends and composites [65]. The rheological analysis was conducted to indirectly assess the effectiveness of the compatibilizing effect of isocyanate on the cellulosic filler incorporated into a biodegradable polymeric-blend matrix. The results of the tests carried out in the oscillation mode for the Mater-Bi/PCL blend used as a matrix and the composites with the constant share of UFC100 modified with different HDI concentrations (1–10 wt%) were presented in the form of storage (G’) and loss (G”) modulus curves as a function of the angular frequency (Figure 7) and complex viscosity curves (Figure 8).

All considered materials, including the blend and the composites containing UFC100 modified with various HDI content, exhibit a similar η*(ω) curve course, i.e., a lack of first Newtonian flow in the low angular frequency range. It is connected with a high amount of thermoplastic starch contained in Mater-Bi, which is used as a matrix; this effect has been previously described for this material in the literature [66,67]. The particles restrict the mobility of polymeric chains and, due to physical interactions with macromolecules, limit their viscous behavior [65]. Complex viscosity values increased significantly after the addition of UFC100; however, with a higher share of HDI, a gradual decrease in viscosity in the entire angular frequency range was noted. This effect is related to cellulose surface modification, reducing its polarity and hydrophilicity [65,68,69], which results in better compatibility of the polymer-filler system, reduced physical interactions between the filler particles dispersed in the polymer matrix, and greater system homogeneity, as was confirmed via SEM analysis. It proves that the filler modification process was properly carried out. The G’(ω) curves analysis allows for an indirect description of the phenomenon of the formation of a physical network of the filler in the molten polymer and exceeding the rheological percolation threshold. In the interlocking of the rigid particles in the polymer melt, the effect of independence G’ of angular frequency changes in the lower ranges is observed [70,71]. While this phenomenon occurs for the non-filled with the UFC 100 blends themselves and is related to the mentioned presence of starch in the Mater-Bi/PCL matrix, it is significantly intensified due to the introduction of cellulosic filler. Interestingly, the range of the limited influence of ω on G’ narrows with the increasing share of isocyanate as a modifier introduced in the thermomechanical and chemical modification process. The composition containing 10 wt% HDI, G’ shows greater values than G”, and for the remaining composites, elastic behavior is dominant in the whole considered angular frequency range and confirms the effect of the physical interaction and mutual contact of the fillers in the molten polymer [70]. Based on rheological measurements, it can be concluded that the addition of 10 wt% of UFC100 significantly deteriorates the rheological properties of the biodegradable blend. However, it is possible to increase the system’s compatibility by using HDI in the filler’s thermomechanical and chemical surface modification process. The interaction’s effectiveness depends on the amount of HDI, and the most favorable rheological and structural effects were noted only for the composite produced with the highest concentration of isocyanate.

### 3.5. Thermal Properties

**Figure 9:**
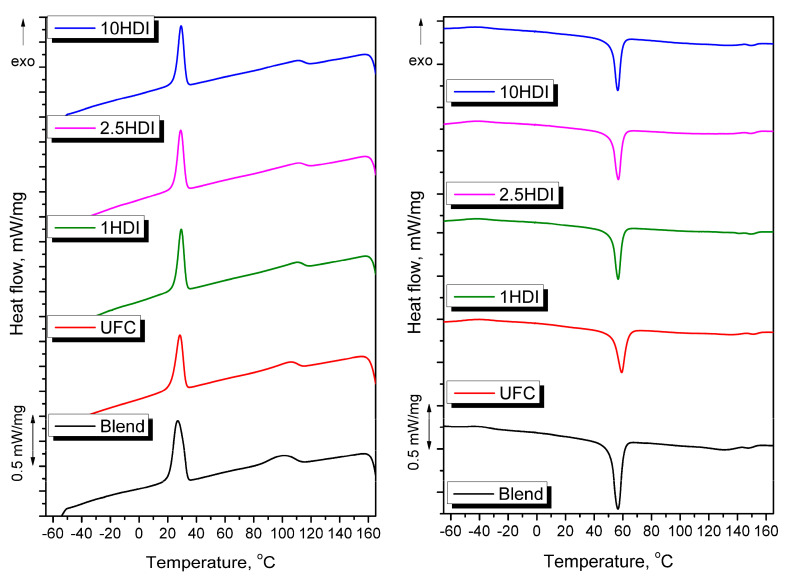
Cooling (**left**) and heating (**right**) thermograms obtained during the DSC analysis of prepared samples (exo ↑).

The differential scanning calorimetry analysis may provide important insights into evaluating polymer composites’ compatibility. Therefore, Figure 9 presents cooling and heating thermograms, while Table 3 provides quantitative data from the prepared sample analysis. In all thermograms, two regions can be distinguished: a low-temperature region (10–70 °C), which is indicative of PCL thermal transitions, and the higher-temperature region (90–155 °C), which is characteristic of PBAT and TPS.

Considering the first one, it can be seen that the melting temperature (T_m_) of PCL was only affected by the presence of as-received cellulose filler, which can be associated with the inferior dispersion of particles resulting from their agglomeration and limited restrictions in the spherulites’ growth [72]. In the case of HDI modifications, particles were finely dispersed, as indicated by the microstructure analysis, which limited the crystallite size and yielded a finer crystalline structure [73]. Melting behavior, particularly the melting enthalpy, provides information regarding the material’s crystallinity. As presented in Table 3, the ΔH_m_ attributed to PCL transition decreased after filler incorporation, which can be mainly attributed to the reduced content of the polymer phase in a composite. However, calculated X_c_ values indicate the decrease in PCL phase crystallinity after the HDI modification of filler particles, which confirms our previous reports [29]. Such an effect could be associated with the interactions between the functional groups of modified fillers and PCL, which limited the mobility of the macromolecules at the interface. Moreover, a reduction in X_c_ was noted despite the increase in crystallization temperature (T_c_), which along with the reduced supercooling parameter (ΔT), points to the nucleating activity of filler particles and enhanced growth rate of the crystalline structure [74].

The higher-temperature region of thermograms is less pronounced and the magnitude of particular peaks is noticeably lower. Considering the melting behavior of the PBAT and TPS phases, similar behavior was noted for PCL. The introduction of as-received cellulose filler noticeably increased T_m_, while filler modification caused its lowering or even disappearance of the signal in the case of the 1HDI and 2.5HDI samples. The highest T_c_ values for PBAT were noted in the samples containing a modified filler, which pointed to the limited movement of macromolecules and facilitated the formation of the crystalline phase.

### 3.6. Thermal Stability

**Figure 10:**
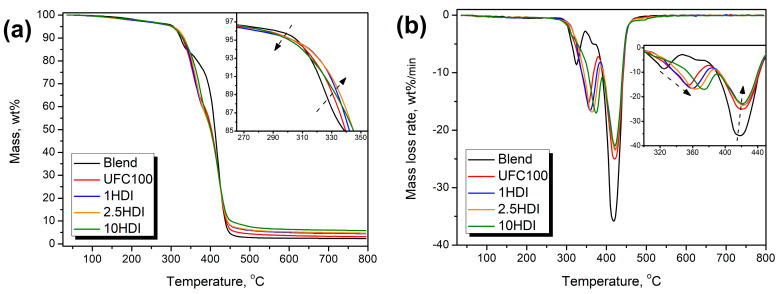
Plots of (**a**) mass loss curves and (**b**) differential thermogravimetric curves visualizing the course of prepared samples’ thermal decomposition.

Thermal stability, along with the abovementioned properties, is also affected by the interfacial compatibility of polymer composites [75,76]. Therefore, it is essential to assess the changes resulting from the fillers’ modifications aimed at interfacial adhesion enhancement. Figure 10 shows that the highest thermal decomposition onset, determined as temperature corresponding to the 2 wt% mass loss, was noted for the unfilled Mater-Bi/PCL blend (210.6 °C), which can be attributed to the exceptional stability of PCL, whose share was reduced after the filler introduction [77]. Similar values of thermal stability have been reported by other researchers for different Mater-Bi grades [35,78]. Considering the course of thermal decomposition, the unfilled blend showed two main steps attributed to the degradation of starch and other polymer components, respectively. The first step can be related to the degradation of starch included in the Mater-Bi material [79]. According to Romagnolli et al. [80], this decomposition step is associated with the breaking of starch chains and the formation of β-(1.6) anhydrous D-glucopyranose and 2-furaldehyde, which are subsequently decomposed at higher temperatures. It can be seen that for the unfilled Mater-Bi/PCL blend, the mass loss during the first degradation step was ~16 wt%, corresponding to the TPS content in the prepared material (20 wt% in Mater-Bi) [35]. The second step was associated with the decomposition of PBAT and PCL, accounting for the remainder of the blend [81,82].

For composites containing as-received and HDI-modified cellulose fillers, a drop in the thermal decomposition onset was noted, which may be associated with the degradation of cellulose starting around 260 °C [83]. The most substantial decrease was noted for samples UFC100 and 1HDI, for which decomposition started in the 190–195 °C range. However, modification of fillers with 2.5 and 10 wt% of HDI shifted the onset to the 202–204 °C range, indicating the efficient enhancement of composites’ compatibility. Similar to the unfilled blend, composites also showed two-step degradation. However, the magnitude of the steps was significantly different. The mass loss noted during the first step was between 38 and 44 wt% and increased with HDI modification. Differences between the composites and unfilled blend were related to the abovementioned decomposition of cellulose filler, which overlapped with the starch decomposition. Moreover, as presented in Figure 2, the introduction of HDI-modified cellulose fillers yielded a generation of covalent bonds at the interface, including urethane linkages, which depending on the structure of isocyanate and alcohol, decompose in the range of 180–300 °C [84]. Therefore, the increasing mass loss during the first decomposition step also confirms the efficient enhancement of interfacial interactions resulting from HDI modification of the applied filler.

## 4. Conclusions

The chemical modification of cellulose filler with HDI noticeably enhanced the compatibility of sustainable composites based on the Mater-Bi/PCL matrix. The analysis of composites’ chemical structure with FTIR spectroscopy pointed to the generation of urethane bonds at the matrix/filler interface, enhancing its quality and reducing filler pull-outs, as indicated by the microscopic observations. The generation of urethane moieties was confirmed by the changes in the thermal decomposition course observed via TGA. Moreover, SEM, along with the results of the rheological analysis, pointed to the limited aggregation of filler particles observed for the UFC100 sample containing as-received cellulose filler. Such behavior was induced by the significant hydrophilicity difference between the applied polymer matrix and the unmodified cellulose particles. Modification with HDI reduced this gap, shifting the surface character towards hydrophobicity, which was expressed by the WCA increase over 90°, putting composites on the edge of hydrophilicity and hydrophobicity. Concluding, HDI modification of cellulose particles should be considered an auspicious direction in the compatibilization of composites based on sustainable polymer matrices as it caused noticeable improvement in the interfacial adhesion.

## Figures and Tables

**Table 1:** Characteristics of applied polymer materials.

Material	Poly(ε-caprolactone)	Mater-Bi
Tradename	Capa 6500	NF803
Producer	Perstorp (Malmö, Sweden)	Novamont SPA (Novara, Italy)
Melt flow index, g/10 min	3.5 (150 °C/5 kg)	7.0 (170 °C/2.16 kg)
Melting temperature, °C	110	58–60

**Table 2:** The parameters describing the roughness of cross-sectional areas of studied samples determined via AFM analysis.

Sample	Whole Image				1 mm^2^ Square Covering the Interface				
	Image Size, µm^2^	Surface Area, 10^3^ µm^2^	R_q_, °	R_a_, °	Surface Area, µm^2^	R_q_, °	R_a_, °	R_sk_	R_ku_
Blend	25	10.1	5.2	3.5	-	-	-	-	-
UFC100		17.3	10.9	8.0	501	9.7	6.9	0.356	5.55
1HDI		21.1	15.7	11.9	732	14.4	10.8	−0.895	4.45
2.5HDI		15.8	11.3	8.6	721	12.5	9.5	−0.765	3.86
10HDI		16.9	12.9	9.6	779	14.0	11.2	−0.714	3.50

**Table 3:** The results of DSC analysis performed for prepared samples.

Sample	PCL					PBAT		TPS
	T_m_, °C	ΔH_m_, J/g	X_c_, %	T_c_, °C	ΔT, °C	T_m_, °C	T_c_, °C	T_m_, °C
Blend	56.6	−23.51	42.13	27.2	29.4	129.5	101.7	147.2
UFC100	59.2	−16.31	41.76	28.5	30.7	135.2	106.0	150.9
1HDI	56.8	−15.11	38.68	29.4	27.4	-	110.5	149.1
2.5HDI	56.9	−15.12	38.71	29.1	27.8	-	111.4	148.8
10HDI	56.5	−14.54	37.23	29.3	27.2	133.8	111.3	149.2

## Data Availability

Data available on request.

## References

[1] Błędzki A.K., Reihmane S., Gassan J. (1998). Thermoplastics Reinforced with Wood Fillers: A Literature Review. Polym. Plast. Technol. Eng..

[2] Olakanmi E.O., Strydom M.J. (2016). Critical Materials and Processing Challenges Affecting the Interface and Functional Performance of Wood Polymer Composites (WPCs). Mater. Chem. Phys..

[3] Hung K.-C., Yeh H., Yang T.-C., Wu T.-L., Xu J.-W., Wu J.-H. (2017). Characterization of Wood-Plastic Composites Made with Different Lignocellulosic Materials That Vary in Their Morphology, Chemical Composition and Thermal Stability. Polymers.

[4] Nurhania N., Syarifuddin S., Armynah B., Tahir D. (2023). Fiber-Reinforced Polymer Composite: Higher Performance with Renewable and Eco-Friendly Plant-Based Fibers. Polym. Renew. Resour..

[5] Mbiada A.A.Y., Musa S., Richter O., Kneer A., Barbe S. (2018). Controlling Surface Hydrophobicity of Cellulose-Lignin Composite Coatings. Polym. Renew. Resour..

[6] Chaturvedi A., Ranakoti L., Rakesh P.K., Mishra N.K. (2021). Experimental Investigations on Mechanical Properties of Walnut Shell and Pine Needle Ash Polylactic Acid Biocomposites. Compos. Theory Pract..

[7] Mohit H., Arul Mozhi Selvan V. (2018). A Comprehensive Review on Surface Modification, Structure Interface and Bonding Mechanism of Plant Cellulose Fiber Reinforced Polymer Based Composites. Compos. Interfaces.

[8] Gholampour A., Ozbakkaloglu T. (2020). A Review of Natural Fiber Composites: Properties, Modification and Processing Techniques, Characterization, Applications. J. Mater. Sci..

[9] Li M., Pu Y., Thomas V.M., Yoo C.G., Ozcan S., Deng Y., Nelson K., Ragauskas A.J. (2020). Recent Advancements of Plant-Based Natural Fiber–Reinforced Composites and Their Applications. Compos. B Eng..

[10] Kalia S., Kaith B.S., Kaur I. (2009). Pretreatments of Natural Fibers and Their Application as Reinforcing Material in Polymer Composites-A Review. Polym. Eng. Sci..

[11] Kabir M.M., Wang H., Lau K.T., Cardona F. (2012). Chemical Treatments on Plant-Based Natural Fibre Reinforced Polymer Composites: An Overview. Compos. B Eng..

[12] George J., Sreekala M.S., Thomas S. (2001). A Review on Interface Modification and Characterization of Natural Fiber Reinforced Plastic Composites. Polym. Eng. Sci..

[13] Vaidya A.A., Gaugler M., Smith D.A. (2016). Green Route to Modification of Wood Waste, Cellulose and Hemicellulose Using Reactive Extrusion. Carbohydr. Polym..

[14] Szefer E., Leszczyńska A., Pielichowski K. (2018). Modification of Microcrystalline Cellulose Filler with Succinic Anhydride—Effect of Microwave and Conventional Heating. Compos. Theory Pract..

[15] Zhang Y., Li H., Li X., Gibril M.E., Yu M. (2014). Chemical Modification of Cellulose by in Situ Reactive Extrusion in Ionic Liquid. Carbohydr. Polym..

[16] Gómez-Fernández S., Ugarte L., Calvo-Correas T., Peña-Rodríguez C., Corcuera M.A., Eceiza A. (2017). Properties of Flexible Polyurethane Foams Containing Isocyanate Functionalized Kraft Lignin. Ind. Crop. Prod..

[17] Musk A.W., Peters J.M., Wegman D.H. (1988). Isocyanates and Respiratory Disease: Current Status. Am. J. Ind. Med..

[18] Geng Y., Li K., Simonsen J. (2005). A Combination of Poly(Diphenylmethane Diisocyanate) and Stearic Anhydride as a Novel Compatibilizer for Wood–Polyethylene Composites. J. Adhes. Sci. Technol..

[19] Ashori A., Nourbakhsh A. (2009). Polypropylene Cellulose-Based Composites: The Effect of Bagasse Reinforcement and Polybutadiene Isocyanate Treatment on the Mechanical Properties. J. Appl. Polym. Sci..

[20] Maldas D., Kokta B.V., Raj R.G., Daneault C. (1988). Improvement of the Mechanical Properties of Sawdust Wood Fibre—Polystyrene Composites by Chemical Treatment. Polymer.

[21] Raj R.G., Kokta B.V., Maldas D., Daneault C. (1989). Use of Wood Fibers in Thermoplastics. VII. The Effect of Coupling Agents in Polyethylene–Wood Fiber Composites. J. Appl. Polym. Sci..

[22] Liew F.K., Hamdan S., Rahman M.R., Mahmood M.R., Lai J.C.H. (2018). The Effects of Nanoclay and Tin(IV) Oxide Nanopowder on Morphological, Thermo-Mechanical Properties of Hexamethylene Diisocyanate Treated Jute/Bamboo/Polyethylene Hybrid Composites. J. Vinyl Addit. Technol..

[23] Hosseinpourpia R., Echart A., Adamopoulos S., Gabilondo N., Eceiza A. (2018). Modification of Pea Starch and Dextrin Polymers with Isocyanate Functional Groups. Polymers.

[24] Kweon D.-K., Cha D.-S., Park H.-J., Lim S.-T. (2000). Starch-g-Polycaprolactone Copolymerization Using Diisocyanate Intermediates and Thermal Characteristics of the Copolymers. J. Appl. Polym. Sci..

[25] Mani R., Tang J., Bhattacharya M. (1998). Synthesis and Characterization of Starch-Graft-Polycaprolactone as Compatibilizer for Starch/Polycaprolactone Blends. Macromol. Rapid Commun..

[26] Ohkita T., Lee S.-H. (2004). Effect of Aliphatic Isocyanates (HDI and LDI) as Coupling Agents on the Properties of Eco-Composites from Biodegradable Polymers and Corn Starch. J. Adhes. Sci. Technol..

[27] Gwon J.-G., Cho H.-J., Lee D., Choi D.-H., Lee S., Wu Q., Lee S.-Y. (2018). Physicochemical and Mechanical Properties of Polypropylene-Cellulose Nanocrystal Nanocomposites: Effects of Manufacturing Process and Chemical Grafting. Bioresources.

[28] Arjmand F., Barmar M., Barikani M. (2012). Isocyanate Modification of Wood Fiber in Enhancing the Performance of Its Composites with High Density Polyethylene. Polym. Renew. Resour..

[29] Hejna A., Kosmela P. (2020). Insights into Compatibilization of Poly(ε-Caprolactone)-Based Biocomposites with Diisocyanates as Modifiers of Cellulose Fillers. Mindanao J. Sci. Technol..

[30] Hejna A., Kosmela P., Mysiukiewicz O., Barczewski M. (2023). Insights into Seawater Biodegradation of Sustainable Mater-Bi/Poly(ε-Caprolactone)-Based Biocomposites Filled with Diisocyanate-Modified Cellulose Particles. Environments.

[31] Hejna A., Barczewski M., Kosmela P., Mysiukiewicz O., Sulima P., Przyborowski J.A., Kowalkowska-Zedler D. (2022). Mater-Bi/Brewers’ Spent Grain Biocomposites—Novel Approach to Plant-Based Waste Filler Treatment by Highly Efficient Thermomechanical and Chemical Methods. Materials.

[32] Hejna A., Barczewski M., Kosmela P., Mysiukiewicz O., Aniśko J., Sulima P., Andrzej Przyborowski J., Reza Saeb M. (2022). The Impact of Thermomechanical and Chemical Treatment of Waste Brewers’ Spent Grain and Soil Biodegradation of Sustainable Mater-Bi-Based Biocomposites. Waste Manag..

[33] Haque M.-U., Alvarez V., Paci M., Pracella M. (2011). Processing, Compatibilization and Properties of Ternary Composites of Mater-Bi with Polyolefins and Hemp Fibres. Compos. Part. A Appl. Sci. Manuf..

[34] Borchani K.E., Carrot C., Jaziri M. (2019). Rheological Behavior of Short Alfa Fibers Reinforced Mater-Bi^®^ Biocomposites. Polym. Test..

[35] Elfehri Borchani K., Carrot C., Jaziri M. (2015). Biocomposites of Alfa Fibers Dispersed in the Mater-Bi^®^ Type Bioplastic: Morphology, Mechanical and Thermal Properties. Compos. Part A Appl. Sci. Manuf..

[36] Bastioli C., Bellotti V., Giudice L., Gilli G. (1993). Mater-Bi: Properties and Biodegradability. J. Env. Polym. Degrad..

[37] Puglia D., Tomassucci A., Kenny J.M. (2003). Processing, Properties and Stability of Biodegradable Composites Based on Mater-Bi^®^ and Cellulose Fibres. Polym. Adv. Technol..

[38] Hejna A., Marć M., Korol J. (2021). Modification of Cellulosic Filler with Diisocyanates—Volatile Organic Compounds Emission Assessment and Stability of Chemical Structure over Time. Nord. Pulp Pap. Res. J..

[39] Hejna A., Marć M., Skórczewska K., Szulc J., Korol J., Formela K. (2021). Insights into Modification of Lignocellulosic Fillers with Isophorone Diisocyanate: Structure, Thermal Stability and Volatile Organic Compounds Emission Assessment. Eur. J. Wood Wood Prod..

[40] Mahović Poljaček S., Priselac D., Tomašegović T., Elesini U.S., Leskovšek M., Leskovac M. (2022). Effect of the Addition of Nano-Silica and Poly(ε-Caprolactone) on the Mechanical and Thermal Properties of Poly(Lactic Acid) Blends and Possible Application in Embossing Process. Polymers.

[41] Gumede T.P., Shingange K., Mbule P., Motloung B. (2022). Miscibility Effect of Biodegradable Aliphatic Poly(Butylene Succinate)/Aromatic Polycarbonate Blends. Polym. Renew. Resour..

[42] Muller L.C., Marx S., Vosloo H.C., Chiyanzu I. (2019). Functionalising Lignin in Crude Glycerol to Prepare Polyols and Polyurethane. Polym. Renew. Resour..

[43] Hansen P.E., Spanget-Larsen J. (2017). NMR and IR Investigations of Strong Intramolecular Hydrogen Bonds. Molecules.

[44] Bukowczan A., Hebda E., Michałowski S., Pielichowski K. (2018). Modification of Polyurethane Viscoelastic Foams by Functionalized Polyhedral Oligomeric Silsesquioxanes (POSS). Compos. Theory Pract..

[45] Celebi H., Ilgar M., Seyhan A.T. (2022). Evaluation of the Effect of Isocyanate Modification on the Thermal and Rheological Properties of Poly(ε-Caprolactone)/Cellulose Composites. Polym. Bull..

[46] Siqueira G., Bras J., Dufresne A. (2010). New Process of Chemical Grafting of Cellulose Nanoparticles with a Long Chain Isocyanate. Langmuir.

[47] Chen Q., Gao Z., Bai L., Xu Z., Gu J. (2021). Water-Dispersible Isocyanate Modified Using Plant-Based Castor Oil: Synthesis and Application as Crosslinking Agent. Ind. Crop. Prod..

[48] Ly B., Thielemans W., Dufresne A., Chaussy D., Belgacem M.N. (2008). Surface Functionalization of Cellulose Fibres and Their Incorporation in Renewable Polymeric Matrices. Compos. Sci. Technol..

[49] Opálková Šišková A., Bučková M., Kroneková Z., Kleinová A., Nagy Š., Rydz J., Opálek A., Sláviková M., Eckstein Andicsová A. (2021). The Drug-Loaded Electrospun Poly(ε-Caprolactone) Mats for Therapeutic Application. Nanomaterials.

[50] Fu Y., Wu G., Bian X., Zeng J., Weng Y. (2020). Biodegradation Behavior of Poly(Butylene Adipate-Co-Terephthalate) (PBAT), Poly(Lactic Acid) (PLA), and Their Blend in Freshwater with Sediment. Molecules.

[51] Aldas M., Rayón E., López-Martínez J., Arrieta M.P. (2020). A Deeper Microscopic Study of the Interaction between Gum Rosin Derivatives and a Mater-Bi Type Bioplastic. Polymers.

[52] Aldas M., Pavon C., Ferri J.M., Arrieta M.P., López-Martínez J. (2021). Films Based on Mater-Bi^®^ Compatibilized with Pine Resin Derivatives: Optical, Barrier, and Disintegration Properties. Polymers.

[53] Laaziz S.A., Raji M., Hilali E., Essabir H., Rodrigue D., Bouhfid R., Qaiss A. (2017). el kacem Bio-Composites Based on Polylactic Acid and Argan Nut Shell: Production and Properties. Int. J. Biol. Macromol..

[54] Tsou C.-H., Ma Z.-L., Yang T., De Guzman M.R., Chen S., Wu C.-S., Hu X.-F., Huang X., Sun Y.-L., Gao C. (2022). Reinforced Distiller’s Grains as Bio-Fillers in Environment-Friendly Poly(Ethylene Terephthalate) Composites. Polym. Bull..

[55] Carvalho A.J.F., Curvelo A.A.S., Gandini A. (2005). Surface Chemical Modification of Thermoplastic Starch: Reactions with Isocyanates, Epoxy Functions and Stearoyl Chloride. Ind. Crop. Prod..

[56] Zhang C., Li K., Simonsen J. (2004). Improvement of Interfacial Adhesion between Wood and Polypropylene in Wood–Polypropylene Composites. J. Adhes. Sci. Technol..

[57] Nam T.H., Ogihara S., Nakatani H., Kobayashi S., Song J. (2012). Il Mechanical and Thermal Properties and Water Absorption of Jute Fiber Reinforced Poly(Butylene Succinate) Biodegradable Composites. Adv. Compos. Mater..

[58] de Campos A., Tonoli G.H.D., Marconcini J.M., Mattoso L.H.C., Klamczynski A., Gregorski K.S., Wood D., Williams T., Chiou B.-S., Imam S.H. (2013). TPS/PCL Composite Reinforced with Treated Sisal Fibers: Property, Biodegradation and Water-Absorption. J. Polym. Env..

[59] Wenzel R.N. (1949). Surface Roughness and Contact Angle. J. Phys. Colloid. Chem..

[60] Li C., Zhang J., Han J., Yao B. (2021). A Numerical Solution to the Effects of Surface Roughness on Water–Coal Contact Angle. Sci. Rep..

[61] Knitter M., Czarnecka-Komorowska D., Czaja-Jagielska N., Szymanowska-Powałowska D. (2019). Manufacturing and Properties of Biodegradable Composites Based on Thermoplastic Starch/Polyethylene-Vinyl Alcohol and Silver Particles. Advances in Manufacturing II: Volume 4-Mechanical Engineering.

[62] Ruggero F., Onderwater R.C.A., Carretti E., Roosa S., Benali S., Raquez J.-M., Gori R., Lubello C., Wattiez R. (2021). Degradation of Film and Rigid Bioplastics During the Thermophilic Phase and the Maturation Phase of Simulated Composting. J. Polym. Env..

[63] Samal S. (2020). Effect of Shape and Size of Filler Particle on the Aggregation and Sedimentation Behavior of the Polymer Composite. Powder Technol..

[64] Duboust N., Ghadbeigi H., Pinna C., Ayvar-Soberanis S., Collis A., Scaife R., Kerrigan K. (2017). An Optical Method for Measuring Surface Roughness of Machined Carbon Fibre-Reinforced Plastic Composites. J. Compos. Mater..

[65] Czajka A., Bulski R., Iuliano A., Plichta A., Mizera K., Ryszkowska J. (2022). Grafted Lactic Acid Oligomers on Lignocellulosic Filler towards Biocomposites. Materials.

[66] Huang H.-X., Zhang J.-J. (2009). Effects of Filler-Filler and Polymer-Filler Interactions on Rheological and Mechanical Properties of HDPE-Wood Composites. J. Appl. Polym. Sci..

[67] Shin B.Y., Lee S., Shin Y.S., Balakrishnan S., Narayan R. (2004). Rheological, Mechanical and Biodegradation Studies on Blends of Thermoplastic Starch and Polycaprolactone. Polym. Eng. Sci..

[68] Nuryawan A., Alamsyah E.M. (2018). A Review of Isocyanate Wood Adhesive: A Case Study in Indonesia. Applied Adhesive Bonding in Science and Technology.

[69] Maldas D., Kokta B.V. (1993). Interfacial Adhesion of Lignocellulosic Materials in Polymer Composites: An Overview. Compos. Interfaces.

[70] Barczewski M., Mysiukiewicz O. (2018). Rheological and Processing Properties of Poly(Lactic Acid) Composites Filled with Ground Chestnut Shell. Polym. Korea.

[71] Li J., Zhou C., Wang G., Zhao D. (2003). Study on Rheological Behavior of Polypropylene/Clay Nanocomposites. J. Appl. Polym. Sci..

[72] Alfonso G.C., Pedemonte E., Ponzetti L. (1979). Mechanism of Densification and Crystal Perfection of Poly(Ethylene Terephthalate). Polymer.

[73] Zhou W. (2011). Thermal and Dielectric Properties of the Aluminum Particle Reinforced Linear Low-Density Polyethylene Composites. Polym. Eng. Sci..

[74] Stachak P., Hebda E., Pielichowski K. (2019). Foaming Extrusion of Thermoplastic Polyurethane Modified by POSS Nanofillers. Compos. Theory Pract..

[75] Chen R.S., Ahmad S., Gan S., Salleh M.N., Ab Ghani M.H., Tarawneh M.A. (2016). Effect of Polymer Blend Matrix Compatibility and Fibre Reinforcement Content on Thermal Stability and Flammability of Ecocomposites Made from Waste Materials. Thermochim. Acta.

[76] Lu N., Oza S. (2013). Thermal Stability and Thermo-Mechanical Properties of Hemp-High Density Polyethylene Composites: Effect of Two Different Chemical Modifications. Compos. B Eng..

[77] Xi X., Jiang G., Wang X., Hu R., Wang R. (2013). Synthesis, Characterization and Degradation Properties of Poly(α-Angelica Lactone-Co-∊-Caprolactone) Copolymers. Polym. Renew. Resour..

[78] Aldas M., Ferri J.M., Lopez-Martinez J., Samper M.D., Arrieta M.P. (2020). Effect of Pine Resin Derivatives on the Structural, Thermal, and Mechanical Properties of Mater-Bi Type Bioplastic. J. Appl. Polym. Sci..

[79] Zdanowicz M. (2020). Starch Treatment with Deep Eutectic Solvents, Ionic Liquids and Glycerol. A Comparative Study. Carbohydr. Polym..

[80] Romagnolli C.M., Leite G.P., Rodrigues T.A., Morelli C.L. (2020). Blend of Cassava Starch and High-Density Polyethylene with Green Tea for Food Packaging. Polym. Renew. Resour..

[81] Nayak S.K. (2010). Biodegradable PBAT/Starch Nanocomposites. Polym. Plast. Technol. Eng..

[82] Mofokeng J.P., Luyt A.S. (2015). Morphology and Thermal Degradation Studies of Melt-Mixed Poly(Lactic Acid) (PLA)/Poly(ε-Caprolactone) (PCL) Biodegradable Polymer Blend Nanocomposites with TiO2 as Filler. Polym. Test..

[83] Stefanidis S.D., Kalogiannis K.G., Iliopoulou E.F., Michailof C.M., Pilavachi P.A., Lappas A.A. (2014). A Study of Lignocellulosic Biomass Pyrolysis via the Pyrolysis of Cellulose, Hemicellulose and Lignin. J. Anal. Appl. Pyrolysis.

[84] Chattopadhyay D.K., Webster D.C. (2009). Thermal Stability and Flame Retardancy of Polyurethanes. Prog. Polym. Sci..

